# Cases of Wounds Penetrating the Thoracic Cavity

**Published:** 1820-10

**Authors:** Baron Larrey


FOR THE LONDON MEDICAL AND PHYSICAL JOURNAL.

### *Cases of Wounds penetrating the Thoracic Cavity.*

By Baron LARREY.

A HORSE-SOLDIER, twenty-two years of age, of a strong constitution and a quiet disposition, was brought to the military hospital *Gros Caillou*, early in the month of February, 1819, in consequence of a severe wound of the chest, which he had received in a duel a few hours previously. The weapon (a horseman's sword), had penetrated the right side of the thoracic cavity to the depth of about three inches, between the fourth and fifth ribs, a little posteriorly to the nipple.

Although the patient had been brought to the hospital, where I received him, without delay, he had lost a great deal of blood, and he was in a state of alarming prostration; nevertheless, there still flowed from the wound, and in pretty considerable quantity, florid and frothy blood; the corresponding region was puffed up by transfusion of air, and the emphysema was already very extensive. The patient had spit blood, his respiration was laborious, the oppression extreme, and he seemed at the point of death. I proceeded to extend the external wound, with all possible expedition, to bring it on a parallel with the intercostal opening, and to favour the escape of the air and blood infiltrated in the cellular tissue. After having, with the intentions just mentioned, applied dry cups on the wound, I brought together its edges immediately by means of stripes of adhesive plaster, the effect of which was favoured by a retentive bandage properly disposed.

This mode of dressing, as I had previously observed many times, arrested, as it were by enchantment, the fatal course of the accidents. The pulse, warmth, and sensitive and organic functions of the patient, which were in a manner extinct, were gradually re-animated and re-established; and in a few hours after every thing announced that the hemorrhage had ceased. The patient now felt himself considerably better. But the presence of the blood effused in the chest soon produced symptoms of irritation, inflammation, and compression, with difficulty of breathing and immobility of the right false ribs. The patient was inclined to lie on the back and side affected. At length, a slight ecchymosis became manifest at the posterior part of the right hypochondre on the third day, which characterized a considerable accumulation. The patient was bled; iced mucilaginous drinks were administered; and several series of cups, with the scarificator, were applied over the whole surface of the chest. These measures appeased the inflammatory symptoms, but those of the effusion remained. The edges of the wound were nevertheless kept in contact, and I insisted on the use of the same measures. On the fifth day having arrived, we could not venture to hope for the removal of the effused fluid by the natural efforts, and I purposed to make a counter-opening in the thorax, if, on the seventh, the symptoms of accumulated fluid continued with the same degree of intensity.

But, in the night of the sixth day, after an access of fever of some violence, which I considered as traumatic, a copious and fetid sweat appeared, which became critical. From this moment the symptoms of the accumulation diminished, and gradually disappeared: the ecchymosis, especially, was completely dissipated, which was to me a certain sign of the absorption of the fluid, which continued to be effected, but in a slow and gradual manner. To aid the natural efforts in this spontaneous process, I applied over the whole of the thoracic region corresponding to the seat of the disease several flying vesicatories, (blistering plasters applied for a short time, so as to produce inflammation of the skin, but not vesication,) and, successively, five cylinders of moxa. All the functions became gradually re-established in this young grenadier; and he left the hospital, in good health, at the end of the third month from his entry.

Several other soldiers of the Guards, affected with similar wounds, and in whom effusion had equally occurred, have recovered under the application of similar means, and without the practice of the operation for empyema.

A sergeant of the Swiss Guards, twenty-one years of age, five feet seven inches in height, of an athletic constitution, was brought to the hospital *Gros Caillou*, the 23d of April, 1819, after having received, in a duel, a wound in the chest by a sword. I was sent for by the medical officer in attendance, and went with all haste to the hospital; and found the patient almost expiring. Two wounds presented themselves; one in the back, of from twelve to fourteen lines in length, situate transversely between the inferior angle of the left scapula and the spine corresponding with the interval between the fifth and sixth ribs; the other, opposite to it, near the left nipple, a little external to it; this was only three or four lines in length, and its direction was nearly transverse. On comparing the longitudinal diameter of these wounds with the breadth of the sword which had produced them, it might at once be believed that the anterior one could not have given passage to half of its blade, since this was of more than an inch in breadth. The patient himself confirmed my conjecture that the sword entered by the back, and that its point had passed out near the nipple, in the space between the sixth and seventh ribs.

The adversary of the patient was obliged to make a great effort to draw out his weapon, the extraction of which was followed by a considerable effusion of blood from both wounds. The Swiss nevertheless walked a few paces, and then fell in a state of syncope, during which he was carried into a neighbouring house, where he recovered his sensibility. A slight dressing was applied to the wounds, and he was then removed to the hospital. He was now pale; there was no pulse in the arteries of the fore-arm; his extremities were covered with cold sweat; his voice was interrupted, and almost extinct; his respiration short and laborious; and his wounds gave vent to florid and frothy blood. The intervals of the wounds were distended by an emphysematous engorgement. The introduction of a probe in the anterior wound, although made in a gentle manner, was accompanied with a sense of anguish and distress without positive pain. I extended largely the exterior orifice of both the wounds, and applied over them and on the surrounding parts of the chest several cups, some with, others without, the scarificator; with the precaution of protecting the wounds from the contact of the external air. I then brought their edges together by stripes of sticking-plaster, and finished the dressing by a retentive bandage. The hemorrhage seemed to be arrested; the patient recovered the power of speech; the warmth of the body was gradually re-established, and its development was favoured by frictions, especially on the trunk, made with very hot oil of chamomile: respiration became more free, the pulse re-appeared in a sensible manner in both the radial arteries; that of the left, however, was less forcible than that of the right.

Orders were given that he should be bled, if symptoms should arise indicative of it; and nitrated chicken-broth prescribed as his only nourishment and drink. The patient was placed in a favourable situation, and committed to the especial care of a medical officer.

The night having been passed in a turbulent manner, and symptoms of re-action and irritation, with spitting of blood, having appeared, the attending surgeon practised a copious blood-letting from the arm, and had two laxative glysters administered.

On the 24th, at my morning visit, the patient suffered severe pains in the course of the anterior wound, with a sense of oppression: he sighed frequently, and was very restless; his cheeks presented spots of redness; his pulse was quick, thirst urgent; and spasmodic motions occurred in the thigh of the side affected with the wound. The bandage was removed from the anterior wound, without disturbing the adhesive plaster; and an emollient cataplasm applied. Several cups, with the scarificator, were put on the hypochondre and abdomen; another venesection was ordered, and the use of iced and nitrated mucilaginous drinks insisted on. A little calm was obtained; yet the danger still appeared very great, and no one ventured to entertain hope of the recovery of the patient. The surgeon-major of the regiment, Dr. Hedellofer, announced him as dead to the colonel, and prognosticated lesion of the heart, or of the pericardium. In the night of the 25th of April, an access of traumatic fever appeared, during which the surgeon on duty practised a third and copious bleeding, which was followed by an abundant sweat, and a favourable state of calmness. On the 28th the dressings were wholly removed: a very little bloody serum oozed from the posterior wound, and as much as would fill a *porringer* and a half (une palette et demie) of a fluid of a more albuminous nature flowed from the anterior wound: this effusion was followed by a subsidence of the chest, greater ease in the respiratory actions, and the return of the sensation of the beating of the heart against the parietes of the chest. All the other symptoms of accumulated fluid, which appeared to me to be limited to a circumscribed space in the thoracic cavity, gradually disappeared.

But, at the same time, severe pain came on, which appeared to rise from the posterior wound to the summit of the shoulder, and to the most lateral part of the left thoracic cavity. Two cupping-glasses, with the scarificator, and an embrocation with camphorated oil of chamomile, dissipated this affection, which I attributed to lesion of some branches of the spinal accessory nerve.

From this moment we conceived great hopes; and indeed the state of the patient improved until the 4th of May, at which period there were still some signs of compression, or of a new collection in the precordial region. A probe carefully introduced gave vent to half a porringer-ful of purulent serum. The beating of the heart, which had again disappeared, was re-established. The pulse in the left arm nevertheless remained smaller, and the whole limb somewhat emaciated. The posterior wound promptly cicatrized: the purulent matter furnished by the anterior became smaller in quantity, and of a good quality; and adhesion of the edges of the wound soon took place. At length, to our great surprise, this young soldier had perfectly recovered his health before the forty-first day from the accident; at which time he went on foot to a court-martial, to which he had been summoned. He left the hospital on the 7th of June, 1819, and was presented at a sitting of the Medical Society a few days afterwards.

At the present time, the 16th of March, 1820, this grenadier, who enjoys in other respects, at least to all appearance, good health, presents the following remarkable circumstances: 1st, the left arm is thinner and weaker than the right; 2d, the wounded side of the chest is sunken, and the nipple is about an inch lower than the right; 3d, the pulsation of the heart is confined and concentrated immediately under the cicatrix of the anterior wound and the spot just surrounding it, as if this organ were attached by its enveloping membrane to the interior of this cicatrix; which seems to confirm the idea we had, that the pericardium, if not the heart itself, had been wounded.

The following case seems to prove the justness of the propositions I have made respecting the mode of dressing wounds penetrating the thoracic cavity. The subject of it is a voltigeur of the sixth regiment of Guards, about twenty-three years of age. This soldier was brought to the hospital *Gros Cailiou*, the 30th of September, 1819, just at night-fall. He was severely wounded in the chest by the thrust of a sword, which he had received in a duel a few hours before he was brought to the hospital. The state of extreme weakness and danger which he was in, induced the medical officer on duty to refrain from disturbing the application which had been made to the wound, which consisted of several handkerchiefs tied round the chest like a girdle. Besides, not perceiving any external hemorrhage, he only interested himself with the use of means calculated to re-establish the almost extinct vital powers of the patient.

On the following morning, at my visit, I discovered, on the right side of the chest, above the nipple, a perpendicular wound, of about an inch in extent, having a backward and inward direction; from which there was effusion of florid and frothy blood, which had moistened all the coverings that had been placed on it. There was very copious expectoration of blood, oppression, short and laborious breathing, pallor of the face and lips; the pulse was small and wiry, the extremities cold; the voice of the patient was interrupted, and hardly sensible: every thing, indeed, seemed to announce the near approach of death. The weapon (which was a narrow sabre) had penetrated into the chest to the depth of about two inches, cutting transversely and throughout the whole of its thickness the cartilage of the third rib, very near its connection with the bony portion. I considered, therefore, that two branches of the intercostal arteries were wounded, the superior lobe of the right lung divided; and, from the convulsive muscular motions which ensued, as a convulsive laugh, and other signs of nervous injury, as peculiar pains, and especially neuralgia, in the arm of the same side, that the phrenic nerve had been wounded in its passage along the surface of the mediastinum.

After I had examined the wound, and ascertained its direction and depth, I lengthened it externally, as much to facilitate the escape of the air diffused in the cellular tissue of part of the chest, as to establish a parallel between its external orifice and the opening in the intercostal muscles. After this extension, there flowed from the chest a great quantity of florid and frothy blood, the evacuation of which was instantly followed by extreme weakness and a very alarming state of suffocation. I closed the wound as quickly as possible by stripes of adhesive plaster, over which a thick compress and a bandage were passed.

Iced mucilaginous drinks, simple emollient glysters, and the most severe abstinence from food, were prescribed. Some minutes afterwards, the pulse, which had been almost extinct before the dressing, became rapidly developed; respiration became more free and deep; the warmth of the body increased in relative proportion; and we saw in a few hours a state of the most imminent danger give place to one which admitted some glimpse of hope, which continued to increase.

On the occurrence of the first degree of violence of re-action, the surgeon on duty fulfilled the conditional order I had given of one or two bleedings from the arm. The same evening, a copious abstraction of blood in this way was followed by the application of several cupping-glasses, without the scarificator, to the side of the chest affected, without disturbing the dressing. The patient was kept in a state of the utmost quietude, and placed on an inclined plane, the head being the most elevated part.

The convulsive motions and the pains in the direction of the nerves, depending on the supposed lesion of the phrenic nerve, already mentioned, constantly existed; and the power of speech, which remained extinct, did not return until a long time after this, and was restored by the means which I shall presently designate. The pains in the right arm and in the principal fingers of this member, were acute and continual. To these symptoms were soon joined those of an accumulation of fluid in the cavity of the chest on the wounded side, with signs of inflammation. I had the venesection repeated several times, and the application of the cupping-glasses was frequently renewed, which were also extended over the shoulder and arm of this side, following especially the course of the nerves of the brachial plexus.

The dressing of the wound was not renewed until the fifth day; and this was effected with all the precautions required to avoid giving access of the external air to the cavity of the chest. All the severe symptoms were successively combatted; and by the ninth day from the accident the patient felt himself sensibly better. He began to speak, and the convulsive or sardonic laugh rapidly diminished. Every thing then announced the establishment of the process for absorption of the effused fluids, and we consequently conceived more confident hopes of the patient's recovery. However, there became manifest, about the eleventh day, some degree of traumatic fever, which disappeared promptly after the application of a vesicatory over the whole of the right hypochondre, and the internal administration of calomel, opium, and the essential salt of cinchona: the calomel was given in rather large doses.

After some days of slight commotion, the state of the patient was again improved, and the advantage continued progressive, so that the patient became convalescent in a few weeks. On the 25th of November, nearly two months from the time of the accident, the cicatrization of the wound was complete, the absorption of the effused fluid very much advanced, and the contraction of the parietes of the right side of the chest had evidently commenced. The process of absorption was favoured by the use of moxas; several piles of which were also applied along the course of the nerves of the brachial plexus, the morbid sympathetic affection of which was not yet wholly destroyed. At length, after two months of most assiduous care, this soldier was completely restored to health; and he left the hospital on the 1st of December, 1819.

